# MINFLUX nanoscopy: Visualising biological matter at the nanoscale level

**DOI:** 10.1111/jmi.13306

**Published:** 2024-04-25

**Authors:** Alexander Carsten, Antonio Virgilio Failla, Martin Aepfelbacher

**Affiliations:** ^1^ Institute of Medical Microbiology, Virology and Hygiene University Medical Center Hamburg Eppendorf Hamburg Germany; ^2^ UKE Microscopy Imaging Facility University Medical Center Hamburg Eppendorf Hamburg Germany

**Keywords:** MINFLUX, single molecule tracking, super‐resolution microscopy

## Abstract

Since its introduction in 2017, MINFLUX nanoscopy has shown that it can visualise fluorescent molecules with an exceptional localisation precision of a few nanometres. In this overview, we provide a brief insight into technical implementations, fluorescent marker developments and biological studies that have been conducted in connection with MINFLUX imaging and tracking. We also formulate ideas on how MINFLUX nanoscopy and derived technologies could influence bioimaging in the future. This insight is intended as a general starting point for an audience looking for a brief overview of MINFLUX nanoscopy from theory to application.

## LAY DESCRIPTION

MINFLUX microscopy can image fluorescent molecules with great precision. This review gives an overview of MINFLUX microscopy from theory to application, explaining how it works, how it has been used in biological studies and how it could be used in the future.

## 1 INTRODUCTION

Fluorescence microscopy has played a prominent role in the natural and life sciences for many decades, as it generally does not require complex sample preparation protocols, but at the same time allows specific visualisation of almost all animate and inanimate matter. Furthermore, during the image acquisition process, the interaction between light and matter can provide a wealth of additional information, among others Raman spectra and pH values.^1^, ^2^ Most importantly, fluorescence microscopy can also be applied to living specimens. Despite all these advantages, fluorescence microscopy has suffered from the technical limitations of the microscopes and fluorescent markers used. Its main limitations are image resolution, detection sensitivity, label quality and image acquisition speed. Many of these factors are entangled; for example, image resolution is strongly dependent on label quality. Since its invention more than 400 years ago, light microscopy has evolved through continuous improvements in image resolution, labels and labelling methods. The first fluorescence‐based microscopy techniques offered a revolutionary approach to visualising biological samples.^3^ Fluorescence microscopy made it possible for the first time to visualise specifically labelled proteins and membrane compartments in cells. However, the first epifluorescence microscopes were unable to resolve objects at distances less than half the excitation wavelength, that is, 300–400 nm, due to the optical limitations of the objectives. Further, this level of resolution could only be achieved if the samples were not too thick, for example, in single cells or nonconfluent cell cultures. Imaging of thicker tissue sections or multicellular layers often resulted in the presentation of huge, shapeless fluorescent blobs. In the 1980s and 1990s, this problem was overcome with the introduction of confocal single or multiphoton laser scanning microscopy.^4^, ^5^ These techniques either use a pinhole in the detection path or exploit the nonlinear optical properties of fluorescent labels for visualising densely labelled samples. Both approaches enabled optical sectioning, that is, optical isolation of tiny slices of the samples, which was the key to improving resolution down to the 200 nm range. Confocal microscopy provided major improvements for visualising the organisation of multilayered cellular structures and for depicting and distinguishing cellular compartments and organelles. However, the detailed visualisation and optical separation of individual biological nanostructures and direct quantification of the interaction of proteins have still not been possible. About 20–25 years ago, a series of revolutionary ideas and technical breakthroughs laid the foundations of modern florescence nanoscopy. The following is a brief overview of these milestones, without following an exact timeline.

One of the first approaches for improving the optical resolution of wide‐field fluorescence microscopy was to use structured illumination patterns to capture multiple images, such as in structured illumination microscopy (SIM).^6^ In the laser scanning microscope technique, on the other hand, optical resolution was improved by capturing the emitted light with an array of detectors (instead of a single PMT/APD detector). This is the principle of image scan microscopy,^7^, ^8^, ^9^ which can achieve the same resolution as a standard confocal microscope with its pinhole almost completely closed. Both approaches can achieve resolutions in the 100 nm range, pushing resolution to the absolute diffraction limit in which the spatial resolution is still limited. All of these techniques have undergone continuous improvement and are still widely used today producing multicolour images with resolutions between 80 and 130 nm. More exciting developments that are able to surpass the absolute spatial resolution came out around the beginning of the 2000s, when the concept of stimulated emission depletion (STED) microscopy and its first experimental implementation were published.^10^ Notably, the concept of STED has already been described in 1994, being the first ‘real’ super‐resolution approach.^11^ Both, STED microscopy and its conceptual extension reversible saturable optical fluorescence transition (RESOLFT) microscopy^12^ are laser scanning based techniques. These approaches are characterised by two key factors: (1) the use of an additional scanning beam to depopulate or discharge the excited state of the fluorescent molecule by a nonfluorescent process such as stimulated emission and (2) the confinement of a region where fluorescence is still dominant is reduced to a small volume of a few cubic nanometres. In particular, STED microscopy uses two scanning beams, a standard one for excitation and a doughnut‐shaped one for inducing stimulated emission, on the fluorescent ring of the doughnut, but not in the doughnut hole. Consequently, the resolution can be improved by shrinking the doughnut hole.

At the same time, studies were carried out to precisely determine the location of a fluorescent object.^13^ This research trend probably inspired the first single molecule localisation microscopy (SMLM) experiments. The first implementation of SMLM was a wide‐field approach using different techniques to selectively activate subsets of the labelling molecules, while the majority of emitters is kept in a nonfluorescent state prior to imaging.^14^, ^15^, ^16^ In this way, the emitting molecules are separated in time and can be visualised and localised individually. The final image consists of the localisation map of all detected molecules. The more precise the localisation, the higher the resolution.

In principle, both STED/RESOLFT and SMLM can achieve single molecule resolution. Therefore, the inventors of these approaches were awarded the Nobel Prize in Chemistry in 2014. However, to date, there are still not many suitable labels that allow this level of resolution to be achieved in a biological sample. In fact, the energy required to produce an emission spot of a few nanometres in STED microscopy still exceeds the maximum amount of light that a fluorescent molecule can withstand without being destroyed, as usually also light from the depletion laser gets absorbed by the dyes, resulting in increased photobleaching.^17^ On the other hand, only rarely and in extreme cases wide field SMLM has been providing a localisation precision of 1–5 nm. The localisation precision σ of wide field SMLM can be represented by the following formula:

(1) $$\sigma \geq \sqrt{\left[\frac{\sigma_{0}^{2}+a^{2}/12}{N}\right]\left[\frac{4}{3}+\frac{8\pi \left(\sigma_{0}^{2}+a^{2}/12\right)b^{2}}{Na^{2}}\right]}$$

and therefore depends on the full width at half maximum of the point spread function (PSF) ($\sigma_{0}$),the camera pixel size *a*, the background intensity *b* as well as the total number of detected photon *N* from an individual molecular emitter.^18^ Commonly used fluorescence molecules have a photon budget of about 10^2^ to 10^6^ photons.^18^, ^19^ These photon budgets combined with detection limitations associated with wide field SMLM normally allow to achieve localisation precision in the range of 15 to 20 nm (Ref. (20) and references therein). Although it is not excluded that in the near future labels will be implemented that can achieve this level of resolution, for the time being, STED and wide‐field SMLM do not allow extensive research on fixed and living biological probes with a resolution below 10–20 nm. However, biological systems such as cells or microbes are densely packed organisms with proteins of only a few nanometres in size and molecular processes and interactions can take place in the low nanometre range. Thus, the resolution achievable with STED or wide field SMLM might still not be sufficient to shed light on research questions in the low nanometre scale.

In this context, the introduction of the minimal photon fluxes (MINFLUX) nanoscopy concept six years ago^21^ and later MINSTED nanoscopy^22^ represents a new revolutionary milestone for super‐resolution light nanoscopy. Briefly, MINFLUX is a laser scanning confocal SMLM approach that uses a structured illuminated light pattern with a strong intensity gradient to localise individual emitters. This structured light pattern can be generated directly by the excitation beam (MINFLUX) or by a combination of excitation and depletion beams (MINSTED). What makes these techniques revolutionary is that the localisation precision of a few nanometres (or even subnanometres) can be achieved with a relatively small number of emitted photons.^21^

The few photons required to localise molecules in the subnanometre range can be regularly emitted from standard fluorescent molecules used to label biological samples.^18^, ^19^ As described in this review, MINFLUX nanoscopy is rapidly evolving as an approach for imaging fluorescent molecules in biological samples at a resolution previously achieved only by electron microscopy (1–10 nm). For the first time fluorescence microscopy is capable to produce images of fluorescent molecules with resolution similar or very close to the ones produced by electron microscopes generating images of electron densities.^23^ This is an important scientific milestone since these two approaches, electron and light microscopy can thus provide complementary information about biological nanostructures, macromolecular complexes and the specific proteins therein. It is hoped that MINFLUX nanoscopy and electron microscopy can be combined in the future as a correlative method to obtain helpful information from the best of both (microscopy) worlds.

It has been suggested that the properties of MINFLUX and MINSTED can also be achieved by wide‐field SMLM. However, as discussed above, this is certainly only possible under optimal conditions (extremely high number of photons emitted by a fluorescent molecule). MINFLUX nanoscopy can currently achieve a ∼10‐fold higher precise localisation of a fluorophore than wide field SMLM.^24^

At present, MINFLUX is still subject to the same limitations as other super‐resolution microscopy techniques, for example, wide‐field SMLM. The search for ideal dyes for the various super‐resolution microscopy technologies will continue and is a central goal of numerous research groups. While searching for the ideal fluorescent label for a given SMLM approach, not only the dyes photon budget but also other physical parameters such as quantum yield and the on‐off duty cycle have to be taken under consideration.^19^ In addition, all the challenges associated with multicolour imaging also apply to MINFLUX nanoscopy.^18^ Indeed the negative correlation between scanning speed and image resolution keeps to be a challenge common to any super‐resolution light microscopy technique: the higher the image resolution the slower the acquisition speed.^25^ Nevertheless, MINFLUX can achieve a high localisation precision of a single emitter in a shorter time frame in comparison to wide field SMLM as high localisation precision needs less detected photons (see Equation 2). In MINFLUX the acquisition time is highly dependent on the size of the region of interest, as in all laser scanning based microscopy approaches. Certainly, the biggest challenge that MINFUX has inherited from all SMLM techniques is the implementation of clear, transparent and unbiased data processing algorithms that transform localisations into reconstructed images.^26^

In this review, we will describe the actual implementation of MINFLUX, its further developed variants and related techniques. Furthermore, we will provide information about labelling techniques and dyes that are developed in the context of MINFLUX. Its current impact on state‐of‐the‐art biological research will be presented by giving an overview of the so far published studies making use of the high spatial and/or temporal resolution of MINFLUX. Last but not least, we will try to foresee which impact MINFLUX will have on biological and biomedical research.

### 1.1 MINFLUX working principle

MINFLUX nanoscopy offers subnanometre localisation precisions on the molecular scale (down to or less than 1 nm).^27^ MINFLUX is an SMLM method that combines the advantages of STED nanoscopy and wide field SMLM. While single fluorophores are switching independently between on‐ and off‐state, their position is precisely targeted by scanning the sample using for instance, a doughnut‐shaped excitation laser beam with an excitation minimum at its centre.^21^ Indeed, the approximately 10‐fold improvement of localisation precision is due to the ability of precisely scanning an individual fluorescence emitter with a properly structured illuminated beam (in this work, we will only consider doughnut shaped excitation beams but other scanning beams are also valid).

In other words, in classical wide field SMLM the single molecule fluorescence patterns pop in randomly and unpredictably while frames are acquired (undeterministic localisation). The localisation of the fluorescence patterns occurs by properly fitting their intensity distributions. The higher the number of emitted photons the higher the signal to noise ratio the more precise is the molecular localisation (Equation 1).

The key factor for the improved localisation in MINFLUX nanoscopy is the a priori knowledge of the position of the excitation beam when the molecule emits photons: MINFLUX is a so called deterministic localisation procedure. Modern laser scanning devices allow to know the position of the excitation beam with subnanometre precision.

In the following, we describe the most common procedure to localise molecules using MINFLUX nanoscopy as previously published in.^27^

**FIGURE 1:**
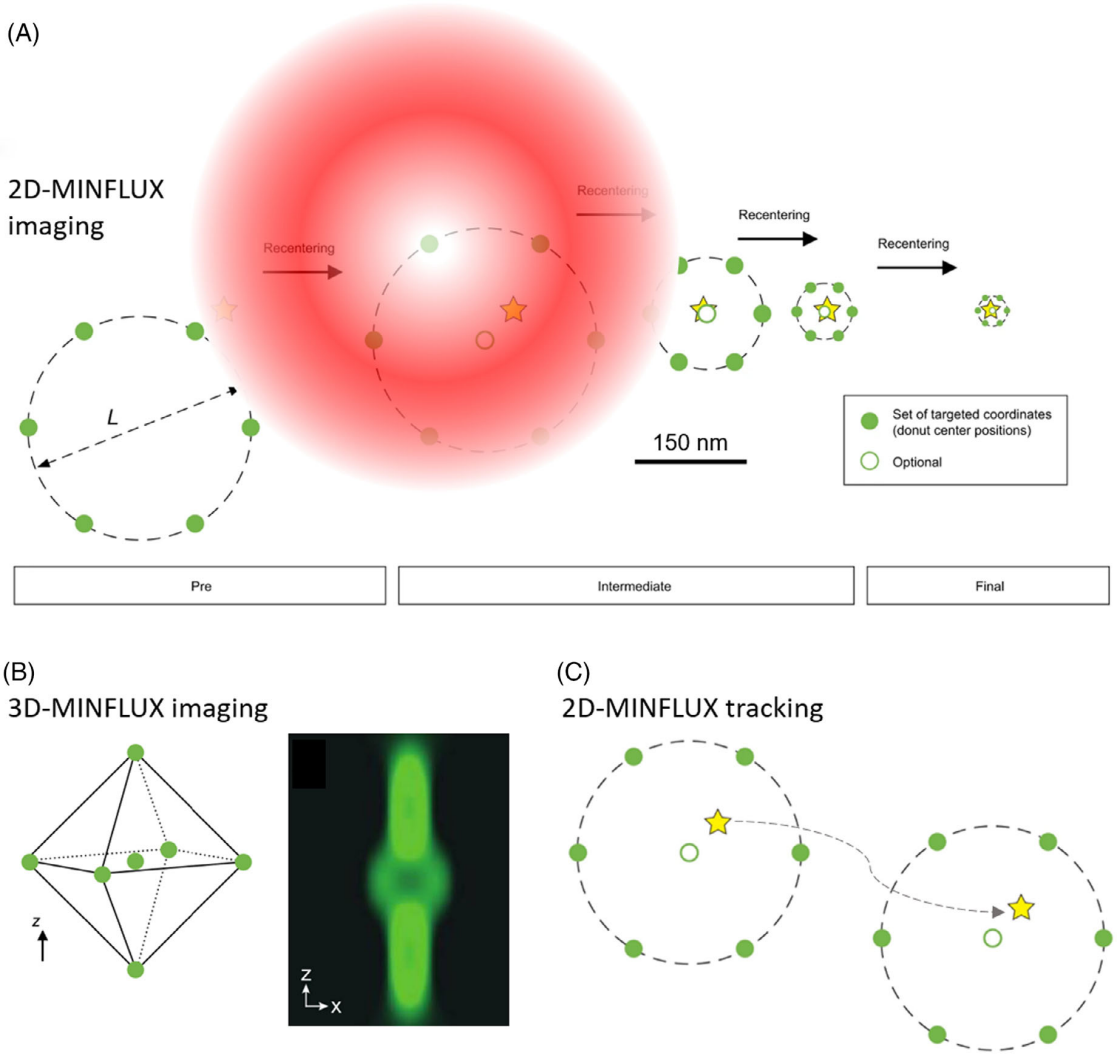
MINFLUX imaging and tracking principles. The green dots represent the targeted coordinates to which the excitation minimum of the doughnut shaped excitation beam is targeted. The yellow stars represent fluorophores in their on‐state. Adapted from Ref. (27). (A) 2D‐MINFLUX imaging. During 2D‐MINFLUX imaging the laser beam, depicted in red, is moved iteratively in a hexagonal pattern towards the fluorophore. After each iteration, the fluorophore can be targeted more precisely based on calculations of the collected photons of the previous iteration. Consequently, the scanning coordinate patterns diameter *L* is reduced and recentred towards the fluorophore in subsequent iterations. (B) 3D‐MINFLUX imaging. During 3D‐MINFLUX imaging first the *xy* position and then the *z* position is targeted during each iteration. Here, the location of the fluorophore is singled out by a bottle‐beam shaped excitation laser guided to coordinated in an octahedronal shape. The bottle‐beam shaped excitation laser is shown in an *xz*‐view. Adapted from Ref. (31). (C) 2D‐MINFLUX tracking. During tracking, the doughnut‐shaped excitation beam is scanned towards a set of coordinates, following the movement of the single fluorescent molecule.

In this configuration, the localisation of a single fluorophore is determined by iteratively positioning the centre of the doughnut‐shaped excitation laser towards the fluorescence emitter. After each iteration, due to the detection of the emitted photons, the location of the fluorescence molecule is better known and the targeted positions of the laser are moved towards the fluorophore. Once the fluorophore is located at the centre of the excitation beam, only a minimum of photons is emitted. Since the central position of the excitation beam is known a priori, fluorescent emitters can be localised with high accuracy using only a relatively small number of photons. Usually in 2D‐MINFLUX a horizontal doughnut shaped excitation beam is targeted in a hexagonal pattern and a centre position towards the fluorophore. 2D‐MINFLUX can achieve a localisation precision in the *xy‐*axis of about 1 nm. Isotropic localisation precisions of around 2–3 nm can be reached in 3D‐MINFLUX by targeting a bottle‐beam shaped excitation laser in an octahedronal shape towards the fluorophore^27^ (Figure 1).

In more detail, MINFLUX localisation results after *k* = 4 localisation steps (Figure 1A) is:

(2) $$\sigma_{4}\geq \frac{16L_{1}}{N_{t}^{2}},$$

$\mathrm{where}\;L_{1}\;$ is the diameter of the region outlined by the targeted coordinate and $N_{t}$ is the total number of detected photon during the entire localisation process (for more details on the MINFLUX localisation precision formula, please refer to Ref. (27)). As a result, the localisation precision scales with $\frac{1}{N_{t}^{2}}$ and not as $\frac{1}{\sqrt{N_{t}}}$ and thus there is an enormous localisation precision enhancement by using MINFLUX versus wide field SMLM acquiring the same number of photons (also compare Refs. (27), (28) and (29)).

The procedure described above can also be used for tracking individual fluorescent molecules that are supposed to be permanently in the excited state.^21^, ^27^ While the 2D/3D localisation procedure is in principle the same, the difference is that, in MINFLUX‐ tracking, the same molecule is localised multiple times while changing its position: the previous localisation is the starting position for the next.^21^, ^27^, ^30^, ^31^

### 1.2 MINFLUX‐related techniques and modalities

After publications describing the proof of principle of MINFLUX and first biological applications, further developments or implementations of MINFLUX have been published. Pulsed Interleaved MINFLUX (p‐MINFLUX) delivers the focus of the doughnut‐shaped excitation via interleaved laser pulses and enables fluorescence lifetime imaging and multiplexing.^32^ Molecular localisation by raster scanning a minimum of light (RASTMIN) is a single‐molecule localisation approach in which ∼1–2 nm localisation precision can be achieved by raster scanning a light pattern comprising a minimum of intensity.^33^ Recently, two‐photon MINFLUX (2p‐MINFLUX) has been described theoretically. 2p‐MINFLUX might require only a quarter of the photons to reach the same localisation precision of single‐photon MINFLUX.^34^ Interferometric MINFLUX (I‐MINFLUX) features two pairs of oblique beams that interfered destructively in the focal plane leading to a higher steepness of the excitation beam, which results in a higher localisation precision using the same number of photons in comparison to traditional MINFLUX. I‐MINFLUX has been used to record the stepping of kinesin‐1 with an impressive spatiotemporal resolution and is further described below.^35^

SIMFLUX is a camera‐based SMLM technique in which the estimated centre of the dye and its relative position with respect to a shifting sinusoidal illumination pattern are used to extract the molecules position. In the first implementation of this approach, applying structured illumination microscopy to SMLM, provided about the doubled localisation precision on DNA‐origami in comparison to DNA‐PAINT and STORM imaging.^36^ Several studies introducing structured illumination‐based point localisation estimator (SIMPLE),^24^ repetitive optical selective exposure (ROSE)^37^ and modulated localisation (ModLoc)^38^ have recently been carried out to further improve the axial resolution of SIMFLUX.

Finally, another laser scanning SMLM techniques that uses a localisation procedure similar to MINFLUX but with a different excitation approach is MINSTED nanoscopy. MINSTED is based on STED and delivers a spatial precision and resolution down to the molecular scale. Here, the centre and thus the minimum of a STED doughnut beam functions as a precisely movable reference coordinate for localisation. A single emitting fluorophore is located by positioning the centre of the STED doughnut exactly above the fluorophore, resulting in a minimal STED effect and thus fluorescent emission.^22^ MINSTED has been proven to enable localisation precisions in the Ångström range.^39^

### 1.3 Labels and fluorophores

In fluorescence microscopy, the fluorescent dyes but not the target structures (e.g., proteins) are directly visualised. Thus, the labels and fluorescent dyes attached to the molecules of interest require special attention to benefit of the exceptionally high resolution and localisation precision achievable with, for example, MINFLUX nanoscopy. A conventional immunofluorescence approach using a primary antibody against the molecule of interest (MOI) and a fluorescently labelled secondary antibody displaces the fluorophore up to around 20–25 nm from the MOI.^40^ In super‐resolution microscopy generally, but in particular with MINFLUX, it should be taken particular care that the fluorophore is brought to the MOI as close as possible. This can be achieved for example by the use of self‐labelling enzymes such as Halo‐ or SNAP‐tag or also nanobodies against the MOI itself or a peptide tag, such as the ALFA‐tag.^41^, ^42^, ^43^ A systematic overview of different fluorescent labels in relation to a structure of interest has been published before.^44^

Several developments and optimisations of advanced labelling approaches have been published in the context of MINFLUX nanoscopy. One of the most intriguing ways to reduce the offset of fluorescent dyes attached to proteins is by labelling the proteins with unnatural amino acids (UAAs) that are introduced using genetic code expansion (GCE). These UAAs harbour ‘clickable’ side chains that can be reacted with fluorophores holding a reactive group, for example, tetrazine (among others^45^). An advanced way to enhance the incorporation of UAAs into POIs has been published in the context of MINFLUX nanoscopy. Here, an optimised pyrrolysyl‐tRNA synthetase is used in combination with an orthogonal tRNA to improve the incorporation efficiency of the UAA. This method has been shown to work in mammalian cells and bacteria and was also used to label U2OS cell filopodia for MINFLUX nanoscopy.^46^

Apart from the choice of labels, another crucial factor is the choice of fluorophores/dyes. As in any SMLM method, also in MINFLUX imaging fluorescent emissions are separated in time. This can be achieved, for example, by photo‐switching, that is, fluorophores which can stochastically change between a bright on‐state and a dark off‐state.^18^ Usually cyanine‐based dyes such as Alexa Fluor 647, CF660C and CF680 are used for this purpose. These dyes have been the first choice for direct stochastic optical reconstruction microscopy (dSTORM) for many years and were successfully used in numerous MINFLUX studies as described below. Nevertheless, these dyes might be well suitable for dSTORM, for example, in terms of their on‐/off‐switching speed and behaviour,^19^ but are not necessarily optimal for MINFLUX nanoscopy.^47^ In addition, specific buffers containing, for example, thiols to induce the on‐/off‐switching of the dyes are required.^48^ These buffer requirements restrict live‐cell imaging applications of SMLM approaches. Furthermore, multicolour SMLM imaging becomes difficult, as the ‘blinking‐buffer’ needs to be adapted to each dye individually. Using multiple fluorophores at the same time results in a compromise buffer that may not have ideal properties for either of the dyes.^49^ Consequently, quantitative analysis might become difficult or lead to unclear results. These challenges and the necessity of small labels stimulate multiple studies on new and innovative fluorescent probes in the context of MINFLUX nanoscopy.

**FIGURE 2:**
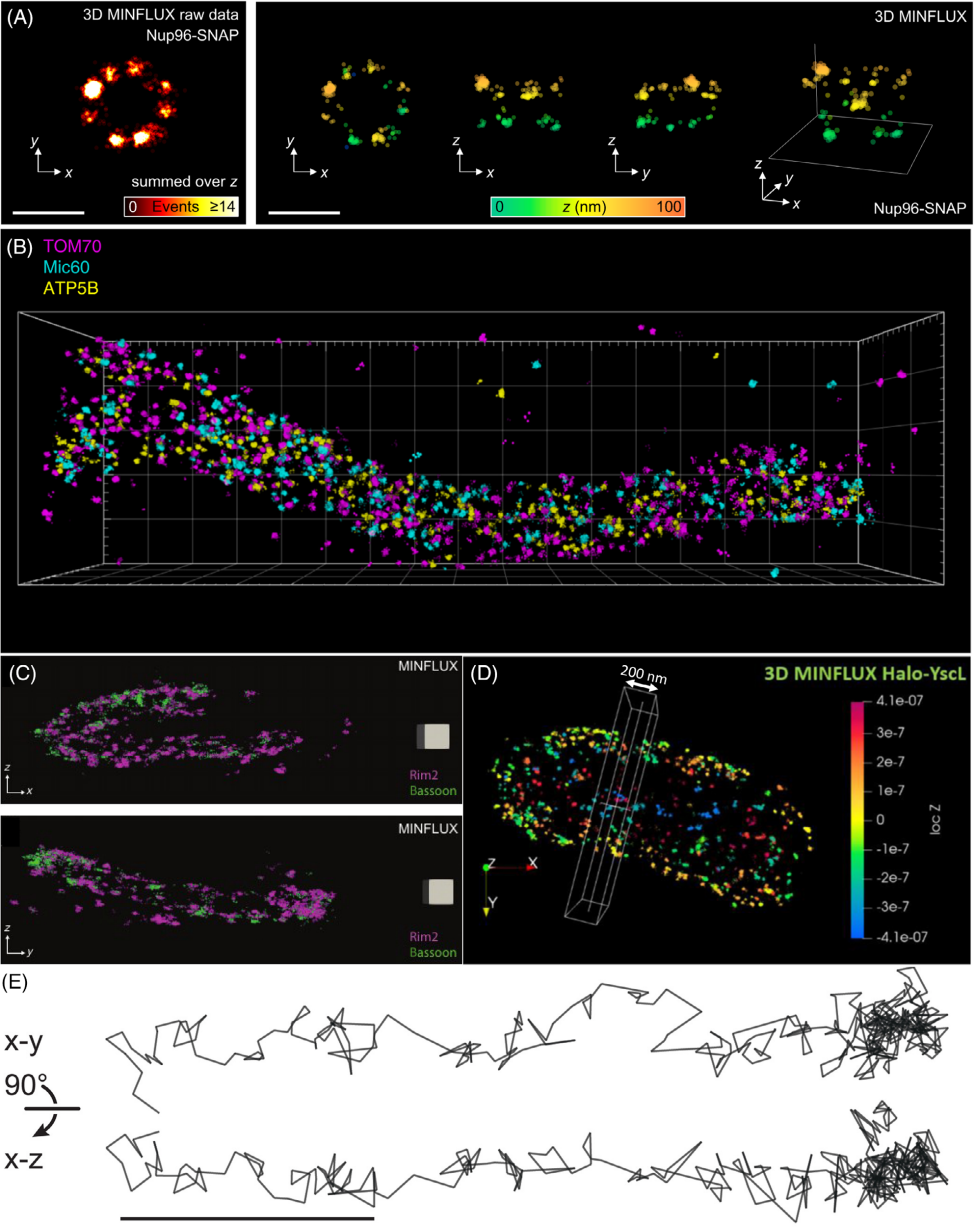
Selection of published MINFLUX imaging and tracking applications in biological samples. (A) *xy*‐ and *xyz*‐view of the SNAP‐tag labelled nuclear pore complex protein Nup96 recorded with 3D‐MINFLUX imaging. Scale bar: 200 nm.^27^ (B) 3D DNA‐PAINT MINFLUX multiplexing of mitochondrial proteins TOM70, Mic60 and ATP5B.^53^ The size of the bounding box is 3.4 × 1 × 0.6 µm^3^. (C) *xz*‐ and *yz*‐views of rod ribbon active zone proteins Rim2 (magenta) and Bassoon (green) stained using 3D‐MINFLUX nanoscopy.^64^ The grey square box has an edge with a length of 100 nm. (D) *xy*‐view of the Halo‐tag labelled type three secretion system component YscL in *Yersinia enterocolitica* using 3D‐MINFLUX nanoscopy. The white boxed area has a depth of 200 nm along the *x*‐axis.^66^ (E) 3D‐MINFLUX tracks of kinesin in live U2OS cells in *xy*‐ and *xz‐* view. Scale bar: 100 nm.^31^

Some of these can be tackled by point accumulation in nanoscale topography (PAINT) technique. In PAINT fluorophores have a freely diffusing and an immobilised state, namely, when binding to a target. Mostly this is achieved via DNA‐PAINT, where dyes are linked to single stranded DNA filaments which are transiently immobilised upon binding to a corresponding DNA strand attached to the target.^50^, ^51^ Additionally, a PAINT approach for the widely used Halo‐tag has been introduced. In contrast to the covalent, irreversible bond that is formed with conventional fluorescent Halo‐tag substrates, the newly developed fluorogenic xHTL probes bind transiently to the Halo‐tag and do not require specific ‘blinking buffers’.^52^ Generally, PAINT approaches do not suffer from massive bleaching problems as the dyes attaching to the target of interest are constantly exchanged. A combination of DNA‐PAINT and MINFLUX has been published and impressively enabled a three colour MINFLUX recording of mitochondria in human cells for the first time (Figure 2B).^53^

Spontaneously blinking fluorophores can also be an optimal choice for MINFLUX nanoscopy. First introduced in 2014,^54^ these silicon rhodamine dyes have recently also been shown to function in MINFLUX nanoscopy with labelled tubulins.^55^ Spontaneously blinking dyes have also been used to strongly accelerate the MINFLUX localisation process. This was achieved with the shortening of ‘on‐times’ of the fluorophore and the optimisation of the MINFLUX localisation routines.^47^ Other groups of markers suitable for MINFLUX nanoscopy have been introduced in recent years. Among them are a series of photoactivatable dyes with hydrophilic carbamate caging groups^56^ and photoactivatable rhodamine, carbo‐ and silicon‐rhodamines emitting in ranges from green to far‐red.^57^ A completely new group of dyes suitable for MINFLUX nanoscopy are photoactivatable xanthones (PaX). These caging‐group‐free fluorophores are described to be highly fluorescent, photo‐ and chemically stable upon irradiation with light.^58^ Also, the PaX dyes have been combined with a tetrazine group to enable selective labelling of UAAs, as mentioned above. This approach combines the strengths of the reduced label size and the beneficial properties of PaX dyes for MINFLUX nanoscopy.^59^

### 1.4 Application of MINFLUX in bioimaging

The publications mentioned above describe impressive inventions, developments and innovations of MINFLUX nanoscopy including various labelling approaches and fluorescent dyes. These technical proceedings have mostly been achieved by imaging ‘*proof‐of‐principle’* samples, like DNA‐Origami or nuclear pore complexes (Figure 2A). However, the purpose of a new microscopy technique is also to give important and meaningful insights into questions throughout multiple fields of life sciences, most often using unique samples to answer specific scientific questions.

Among the first published data using MINFLUX on a biological sample were images of the mitochondrial contact site and cristae organising system (MICOS) complex protein. The MICOS complex is an important part of the mitochondria cristae biogenesis. 3D‐MINFLUX demonstrated that one of its proteins, Mic60 is displaced along two narrow opposite distribution bands in Mic10 knockout cells, whereas in wild type cells it is distributed in a stripe‐like pattern. Also on the basis of these data, the authors suggested that this specific localisation phenotype is an important structure providing element of mitochondria and that the expression level of Mic10 influences the distribution of Mic60.^60^ In a follow up work, 2‐colour 3D‐MINFLUX has been employed to visualise the localisation of Mic60 in relation to Mic10 and Mic19, two other proteins of the MICOS complex. Statistical analysis showed that Mic60 and Mic19 are usually in close proximity to each other, while Mic60 and Mic10 are less spatially correlated. Multiple Mic60 molecules were shown in ring like arrangements with a diameter of ∼50 nm, from which the authors suggest that cristae junctions are surrounded by Mic60. In this publication, the authors also presented a three‐step density‐based clustering algorithm that assigns MINFLUX localisations to individual molecules in an improved manner, compared with a single use of DBSCAN.^61^, ^62^

2D‐MINFLUX has been used to observe interactions between fibronectin leucine‐rich repeat 2 (FLRT2) proteins that are essential for fibronectins central role in tissue development.^63^ It has been known that *in trans* interactions between FLRTs trigger the formation of signalling complexes but it has not been known that FLRTs interact *in cis* at the surface of the same cell. Potential cis‐interactions between two FLRT2 proteins could be observed with a highest probability distance of 6–12 nm between the SNAP‐labels attached to the two FLRT2 proteins. A structural model of the interacting FLRT2 ectodomains with SNAP‐tags based on previously published structural data suggested a distance of ∼8.7 nm between the two fluorophores fitting well to the distances observed by MINFLUX nanoscopy.^63^

Furthermore, 3D‐MINFLUX nanoscopy enabled to resolve the molecular topography of the presynaptic active zone of rod photoreceptors. This was facilitated by a novel sample immobilisation technique named heat‐assisted rapid dehydration (HARD), wherein a thin layer of rod synaptic terminals (spherules) was transferred onto glass coverslips from fresh retinal slices. In this work, all proteins were labelled with an immunofluorescence approach using primary and secondary antibodies. Not only could the convoluted topography of the rod ribbon active zone be resolved, but it could also be shown that the proteins RIM2, bassoon, ubMunc13‐2 and Ca_v_1.4 α1F subunit are organised in two parallel rows. Also distances between the two rows for each of the proteins could be determined. Two‐colour MINFLUX of bassoon and RIM2 showed a colocalisation between those proteins (Figure 2C). From these data, the authors concluded that all the above mentioned proteins form a molecular complex repeating longitudinally on both sides of the ribbon.^64^

MINFLUX nanoscopy also helped to start uncovering activation of PIEZO1 mechanosensitive ion channels in a native environment. The extensive blades of transmembrane domains that form these channels were found to be significantly expanded at rest by bending stress of the plasma membrane. By using chemical and mechanical modulators of the ion channel, the authors could show that the blade expansion and channel activation are correlated.^65^

**FIGURE 3:**
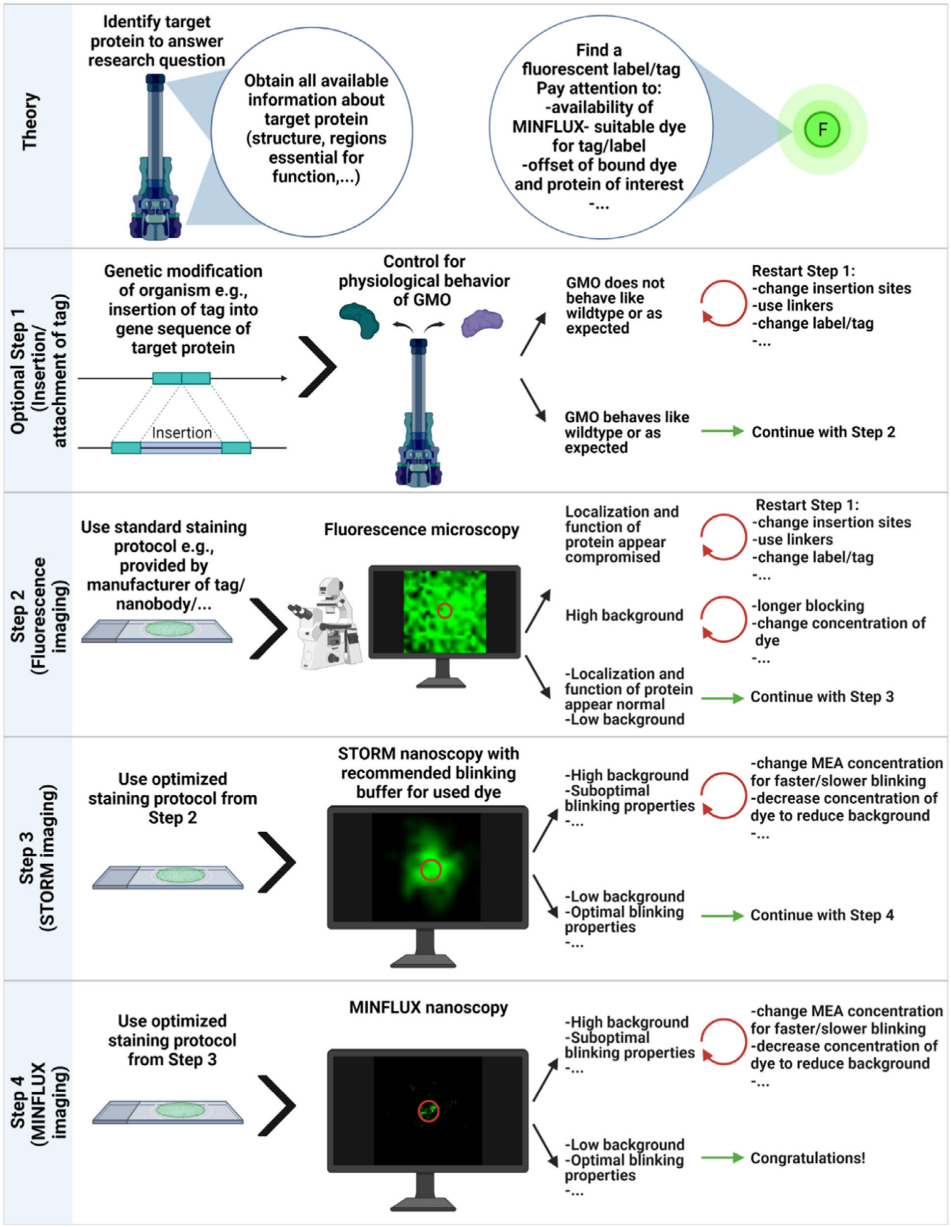
Workflow to guide researchers towards MINFLUX imaging of single molecules in cells. The workflow is exemplary for a microbiological research question. Adjustments might be necessary especially for Step 1 (Genetic modifications). GMO: genetically modified organism; MEA: mercaptoethylamine. Created with BioRender.com. *Source*: Ref. (66).

In one of the first publications on MINFLUX, its single molecule tracking modality has been impressingly demonstrated on diffusing 30S ribosomal subunits in living *Escherichia coli*.^21^ First MINFLUX imaging data in bacteria were published on type three secretion system (T3SS) components in *Yersinia enterocolitica*.^66^ The dynamic T3SS sorting platform component YscL was labelled with a Halo‐tag and could be visualised at the actual size of the protein complex. 3D‐MINFLUX enabled the visualisation of this complex within a whole bacterium with an isotropic localisation precision of approximately 5 nm (Figure 2D). Furthermore, the T3SS translocon protein YopD was tagged with an ALFA‐tag and fluorescently labelled nanobodies. This approach allowed the visualisation of what was considered single YopD proteins and permitted an estimation of the translocon size in *Y. enterocolitica* to 28 nm.^66^ To help scientists who are interested in performing MINFLUX nanoscopy and guide them from their research question towards a high‐quality MINFLUX image, a workflow for this purpose was included in the latter publication and is now provided in Figure 3.^66^

### 1.5 MINFLUX tracking

MINFLUX nanoscopy is not only an outstanding imaging technique but also enables single molecule tracking (SMT) with a very high temporal and spatial resolution.

Besides the first MINFLUX tracking results described above,^21^ further tracking experiments on lipid diffusion in a supported lipid bilayer^27^ and thermally fluctuating DNA strands^30^ in model systems have been performed. In the latter study, localisation precisions of ∼2 nm with only 400 µs measure time were achieved.^30^

MINFLUX tracking applied for solving biological questions has been used in two back‐to‐back publications and showed the stepping motion of the motor protein kinesin‐1 on microtubules in vitro and in living cells.^31^, ^35^ Indeed, Deguchi and colleagues established 2D and 3D MINFLUX tracking of kinesin‐1 proteins added to previously fixed cells (‘Motor‐PAINT’) and also native kinesin‐1 in living cells on a commercially available MINFLUX microscope.^31^ The kinesins were marked with self‐labelling enzymes at their C‐ or N‐termini. Depending on the position of the label, 8 or 16 nm steps of the kinesin‐1 could be observed and resolved (Figure 2E). Notably, for 3D MINFLUX tracking in live cells a spatial resolution of 3.9 nm and a temporal resolution of 3.0 ms was achieved which also allowed to resolve the kinesins 16 nm steps in 3D.^31^ Moreover, Wolff and colleagues introduced an interferometric MINFLUX approach, as described above.^35^ Only ∼20 photons were sufficient to achieve a localisation precision of about 1.7 nm for an immobilised fluorophore. Using this novel MINFLUX set‐up, kinesin movements could be recorded in one dimension with up to 1.7 nm/ms spatiotemporal precision. Besides the expected steps of ∼8 nm also substeps of ∼4 nm could be observed. Under physiological ATP concentrations the authors could regularly observe 6 and 10 nm steps, and these slightly diverging results are probably due to the size of the label and its position on the kinesin. Additionally, the authors performed further experiments finding out that ATP binds to kinesin in its one‐head‐bound state and that ATP hydrolyses in the 2HB state. All in all, these data show that MINFLUX tracking, with its exceptional spatiotemporal resolution, can already answer open research questions.^35^

In order to give a qualitative idea about the time resolution that can be achieved using a commercial MINFLUX setup it can be considered that a localisation cycle is accomplished in 1 ms. This time can be shortened further, but this results in a reduced localisation precision. Second, a full localisation procedure requires four localisation steps, which could also be reduced further, but this again results in a diminished localisation precision. Thus, it will not be possible to track a molecule that moves more than ∼180 nm (half width of the probing doughnut beam) in the time frame of a full localisation procedure. In other words, it would be difficult to track molecules with higher speed than ∼45 nm/ms. As mentioned above, adapting the localisation sequences can increase these limits.

### 1.6 Outlook

MINFLUX brought both imaging and tracking to a new level. Using MINFLUX imaging, molecules can already be detected with a precision down to their actual size on a regular basis. In the future, the developments of new tags, labels and dyes will further improve MINFLUX nanoscopy. Indeed, to exploit the full potential of MINFLUX imaging, it is crucial to bring the fluorophore as close as possible to the target. Self‐labelling enzymes and nanobodies are already a good step towards that direction, especially because they are broadly applicable.^44^ Anyhow, at the moment, the use of unnatural amino acids reflects the ideal labelling situation, but at present, they are not straight‐forward to handle and harbour the danger of increased background staining (an unideal situation to any kind of imaging, especially SMLM approaches). Background fluorescence affects the localisation accuracy in all SMLM approaches. It can also affect MINFLUX microscopy due to the comparatively large area of the MINFLUX excitation donut during the localisation procedure, also if the MINFLUX localisation procedure occurs in few milliseconds. To our knowledge, there are so far no quantitative studies about the impact of background fluorescence in MINFLUX microscopy. Further developments and innovations concerning the dyes will improve MINFLUX imaging, especially for multicolour MINFLUX. PAINT approaches circumvent some of the problems present with multicolour MINFLUX and other SMLM techniques but may also have some drawbacks, such as the increased imaging time.^67^ Also here, first developments are being made, for example, the above mentioned PaX dyes.^58^ Notably, developments and innovations in labelling approaches can also improve the localisation precision itself, as impressingly shown recently by a new proof‐of‐principle PAINT implementation called ‘resolution enhancement by sequential imaging’ (RESI), in which orthogonal DNA sequences are sequenced sequentially. Such developments widen also the labelling approaches applicable for MINFLUX.^20^

The first MINFLUX tracking applications show the power of MINFLUX and give researchers an idea to which level of detail molecules can be followed. Although the molecules diffusion speed might be a limitation for MINFLUX tracking, the interferometric MINFLUX introduced be Wolff and colleagues already demonstrated that such technical limitations can be overcome in the future.^35^ Multicolour MINFLUX tracking remains yet to be developed, but it can be assumed that this will just be a matter of time. MINFLUX tracking will also benefit from improvements in labels and fluorophores. Especially cell‐permeable photo‐stable fluorophores will help to observe molecular processes in the most physiological manner.

In this review, we have described that MINFLUX nanoscopy allows researchers to see what could not be seen before with a fluorescence based microscope. Tremendous progress has already been made to provide the scientific community with user friendly equipment, labels and dyes to answer their research questions.

We would like to conclude this review with a general but still valid consideration. The same principles that apply to all microscopy approaches also apply to MINFLUX nanoscopy: to obtain a meaningful image, an optimal sample has to be provided. To help researchers obtaining an optimal MINFLUX sample, we include here a generally applicable workflow guiding researchers from an initial research question to MINFLUX imaging (Figure 3).^66^ Finally, if an optimal image has been acquired, a clear, justified and an easily understandable set of procedures has to be defined in order to process the data in a transparent and unbiased way.

## CONFLICT OF INTEREST STATEMENT

The authors declare no conflict of interest.
